# Physiological and Endogenous Hormonal Responses During In Vitro Culture of Citral-Rich *Camphora officinarum*

**DOI:** 10.3390/plants15213497

**Published:** 2026-11-09

**Authors:** Xiao Zhou, Xingge Huang, Shengyu Guo, Yunzhu Chen, Peiwang Li, Qiang Liu, Changzhu Li, Mei Chen, Wenbin Zeng, Yanju Song, Jingzhen Chen, Yan Yang

**Affiliations:** 1 State Key Laboratory of Woody Oil Resources Utilization, Hunan Academy of Forestry, Changsha 410018, China; zx924647234@163.com (X.Z.); cyzcarol@foxmail.com (Y.C.); lindan523@163.com (P.L.); lichangzhu2013@aliyun.com (C.L.); 2 College of Life Science and Technology, Central South University of Forestry and Technology, Changsha 410004, China; hxg4940@163.com (X.H.); liu.qiangcs@163.com (Q.L.); csuft85658537@163.com (W.Z.);; 3 Department of Economics and Management, Changsha University, Changsha 410022, China; guosy2016@sina.com; 4 Henan Jigongshan National Nature Reserve Affairs Center, Xinyang 464133, China; chenyujunhe@126.com; 5 Key Laboratory of National Forestry and Grassland Administration on Utilization Science for Southern Woody Oilseed, Hunan Academy of Forestry, Changsha 410018, China

**Keywords:** citral-type *Camphora officinarum*, tissue culture, shoot proliferation, adventitious root formation, physiological parameters, endogenous hormones

## Abstract

Bud-bearing stem segments of citral-rich *Camphora officinarum* were used to optimize in vitro shoot proliferation and adventitious root induction by screening basal media and plant growth regulator types and concentrations. Physiological and endogenous hormonal responses were compared between the two culture stages. At each stage, plantlets cultured on the selected medium and a control medium without plant growth regulators were sampled on days 0, 7, 14, 21, and 28. Soluble sugar, soluble protein, and total phenolic contents; superoxide dismutase (SOD) and peroxidase (POD) levels; and zeatin riboside (ZR), gibberellic acid (GA_3_), indole-3-acetic acid (IAA), and abscisic acid (ABA) contents were determined. These parameters were also compared among medium formulations. Murashige and Skoog (MS) medium supplemented with 2.00 mg/L 6-benzyladenine (6-BA), 0.20 mg/L 1-naphthaleneacetic acid (NAA), and 0.05 mg/L kinetin (KT) yielded a proliferation coefficient of 8.16. Within the concentration ranges tested, 6-BA had a greater effect on proliferation than KT or NAA. Half-strength MS medium (1/2MS) was suitable for rooting, and 0.50 mg/L indole-3-butyric acid (IBA) produced a rooting rate of 92.22%. Under the selected culture conditions, soluble sugar content generally increased during shoot proliferation, soluble protein content peaked on day 14, and SOD and POD levels peaked on day 21. During rooting, soluble sugar content declined after day 7, whereas soluble protein content and SOD levels reached high values on day 14. During the middle and later culture periods, treated plantlets generally had higher IAA and lower ABA contents than the controls at both stages. Comparisons among formulations showed that the treatment favorable for proliferation had relatively high soluble sugar and ZR contents, whereas the treatment favorable for rooting had relatively low contents of both. This study identified suitable proliferation and rooting conditions for the genotype represented by the single donor tree examined and revealed differences in sugar dynamics and endogenous hormonal responses between the two culture stages. The selected growth regulator concentrations were suitable within the tested ranges for this genotype. Their applicability to other *C. officinarum* genotypes requires further validation.

## 1. Introduction

*Camphora officinarum* is an evergreen tree in the Lauraceae, and its citral-rich germplasm has potential for aromatic resource development [1]. Studies of leaf essential oils in *C. officinarum* and the closely related *Cinnamomum bodinieri* have revealed differences in oil composition among plant materials, highlighting the importance of germplasm selection and propagation for aromatic resource utilization [1,2]. Vegetative propagation of selected citral-rich trees can increase the availability of planting material for germplasm conservation, cultivation trials, and further utilization. For this purpose, a culture system must support both shoot multiplication and the production of rooted plantlets from the selected donor material.

In vitro culture can produce complete plantlets through axillary bud initiation, shoot proliferation during subculture, and rooting. Culture performance is influenced by the condition of the starting material, the basal medium, and the combination and concentrations of plant growth regulators. Previous work on *Camphora officinarum* established direct organogenesis from nodal segments of embryo-cultured seedlings, with axillary buds removed before adventitious bud induction and regenerated shoots subsequently rooted on hormone-free MS medium [3]. This approach demonstrated regeneration from juvenile seedling material, but differs from the propagation of bud-bearing stem segments collected directly from a selected donor tree. In the citral chemotype of *Cinnamomum bodinieri*, medium screening and transcriptomic analysis identified suitable conditions for IBA-induced rooting and associated changes in auxin-signaling and carbohydrate-metabolism genes [4]. These studies provide useful starting points, but neither establishes the suitability of the same formulations for both proliferation and rooting of the donor material examined here.

Beyond medium selection, physiological and hormonal measurements can help characterize the responses accompanying successful propagation. Studies of *Gardenia jasminoides* tissue culture and rooting in tissue-cultured persimmon shoots have examined culture performance alongside endogenous hormone contents, providing insight into plant responses to exogenous plant growth regulators [5,6]. In woody cuttings, studies of *Acer mono* and *Eucommia ulmoides* have related rooting responses to changes in endogenous hormones and enzyme activities under different treatments or in different varieties [7,8]. These findings support examining physiological status alongside culture performance, but differences in propagation system, plant material, and sampling stage limit direct comparisons. Soluble sugar and protein contents, SOD and POD measurements, and total phenolic content provide complementary descriptions of the cultured material; none alone identifies the process responsible for a growth response.

For the selected citral-rich *C. officinarum* material, the unresolved questions concern which conditions support proliferation and rooting, how physiological and hormonal profiles change during each stage, and whether their associations with culture performance are similar between stages. Time-course observations describe changes during culture, whereas comparisons among formulations reveal treatment differences at a common sampling time. Combining these approaches allows temporal responses under a selected formulation to be distinguished from associations across formulations. This distinction is necessary because a parameter that increases during development need not be highest in the treatment with the best culture performance.

In this study, material from a selected citral-rich *C. officinarum* tree was used to establish aseptic cultures and screen suitable conditions for shoot proliferation and adventitious root induction. The specific objectives were to identify suitable basal media and plant growth regulator formulations, characterize physiological and endogenous hormonal changes during proliferation and rooting, and compare the associations of these parameters with the proliferation coefficient and rooting rate across formulations. The study was designed to support propagation of the tested material and identify stage-associated responses for further investigation, rather than establish causal mechanisms of hormone action.

## 2. Results

### 2.1. Effects of Basal Media and Plant Growth Regulators on Shoot Proliferation

**Figure 1:**
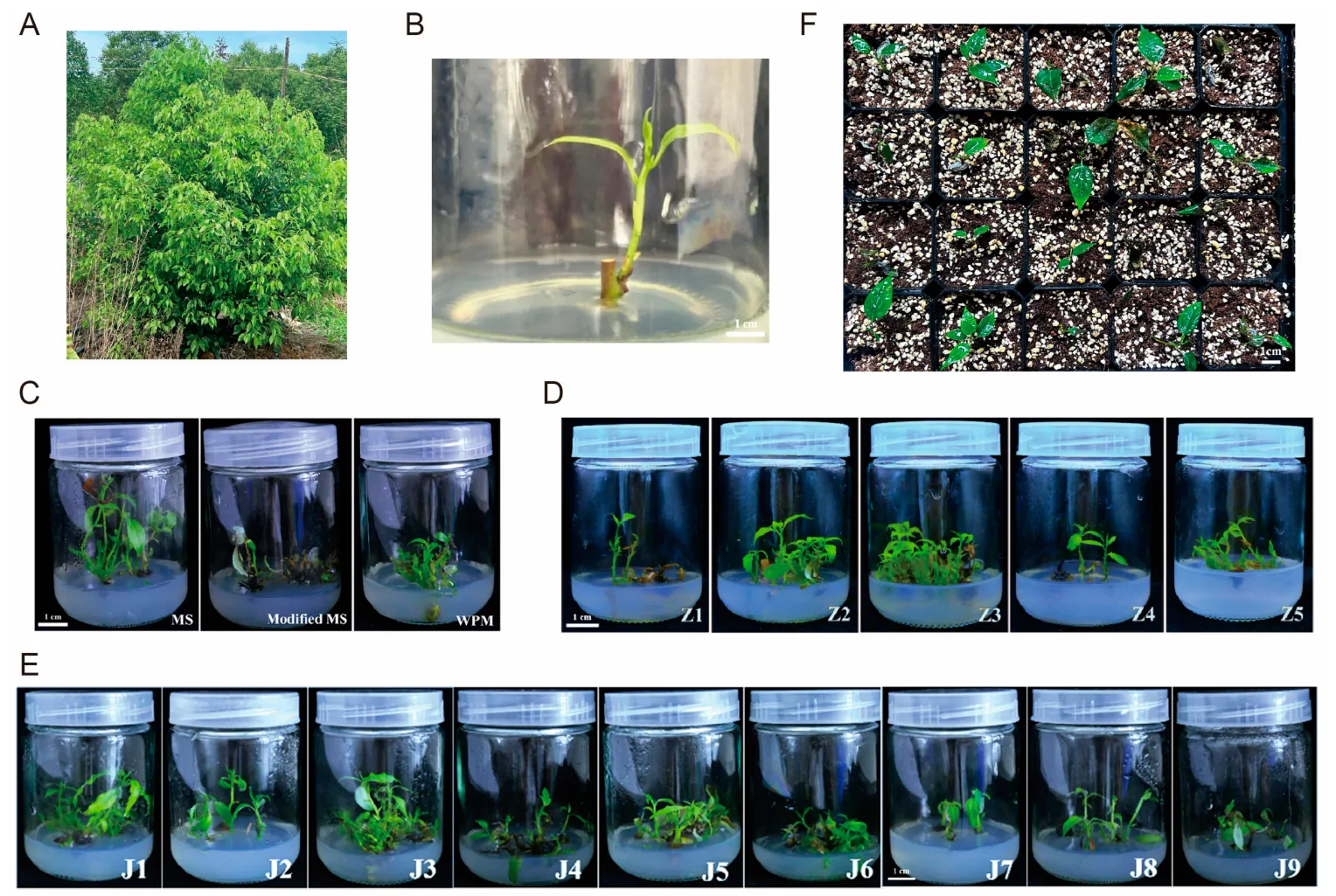
Donor tree, in vitro culture, and growth after transplanting of *Camphora officinarum*. Note: (**A**) Donor tree; (**B**) shoots during initial culture; (**C**) shoot proliferation on different basal media; (**D**) shoot proliferation under different plant growth regulator combinations; (**E**) shoot proliferation under orthogonal treatments J1–J9, arranged from left to right; and (**F**) plantlets transplanted into a 1:1 (*v*/*v*) mixture of peat and perlite. In panel (**C**), MS, Modified MS, and WPM denote MS, modified MS, and woody plant medium, respectively. The Z1–Z5 formulations in panel (**D**) are described in Section 2.3, and the J1–J9 formulations in panel (**E**) are listed in Table 3. Scale bars are indicated in the individual photographs.

Surface disinfection and axillary bud initiation produced aseptic shoots from bud-bearing *C. officinarum* stem segments for subsequent proliferation. In the HgCl_2_ exposure-time experiment, treatment with 75% ethanol for 30 s followed by 0.1% HgCl_2_ for 7 min yielded an explant survival rate of 78.89%. In the IBA concentration experiment for axillary bud initiation, MS supplemented with 2.00 mg/L 6-BA and 0.05 mg/L IBA yielded survival and bud break rates of 61.11% and 55.56%, respectively. Detailed screening results are presented in Tables S1 and S2, and shoot growth during initial culture is shown in Figure 1B.

Under the same plant growth regulator conditions, MS medium produced a proliferation coefficient of 4.57 and a mean plantlet height of 1.79 cm, both significantly higher than those obtained on modified MS or WPM. The proliferation coefficients on modified MS and WPM were 1.73 and 2.03, respectively, and the corresponding mean plantlet heights were 1.24 and 1.18 cm. Neither parameter differed significantly between these two media (Table 1). Shoots grew well on MS, whereas some leaf browning and weak shoot growth were observed on modified MS and WPM (Figure 1C).

Among the five plant growth regulator combinations tested on MS medium, Z3, containing 6-BA, NAA, and KT, produced the highest proliferation coefficient and mean plantlet height (6.37 and 2.01 cm, respectively), both significantly higher than those obtained with the other combinations. Z2, containing only 6-BA and NAA, produced a proliferation coefficient of 3.97, significantly higher than those of Z1, containing 6-BA and IBA, and Z4 and Z5, containing 6-BA and NAA supplemented with ZT and GA_3_, respectively. At the concentrations tested, adding KT significantly increased the proliferation coefficient relative to Z2, whereas adding either ZT or GA_3_ significantly decreased it (Figure 1D; Table 3).

The concentrations of 6-BA, NAA, and KT were further screened using the orthogonal experiment. J1 produced the highest proliferation coefficient (8.16), followed by J3 (5.18) and J5 (4.89). Mean plantlet heights were relatively high in J1 and J3, at 2.73 and 2.66 cm, respectively. J2 and J4 had low proliferation coefficients (1.53 and 1.42, respectively), weak shoot growth, and some wilting (Figure 1E; Table 3). Range values for 6-BA, KT, and NAA were 4.24, 1.71, and 1.51, respectively, indicating that their effects on the proliferation coefficient ranked as 6-BA > KT > NAA within the concentration ranges tested. ANOVA further indicated that all three factors significantly affected the proliferation coefficient (*p* < 0.05; Table S3). Considering both proliferation and shoot growth, the J1 formulation, MS supplemented with 2.00 mg/L 6-BA, 0.20 mg/L NAA, and 0.05 mg/L KT, was selected for the shoot proliferation time-course experiment.

### 2.2. Dynamics of Physiological Parameters and Endogenous Hormones During Shoot Proliferation

**Figure 2:**
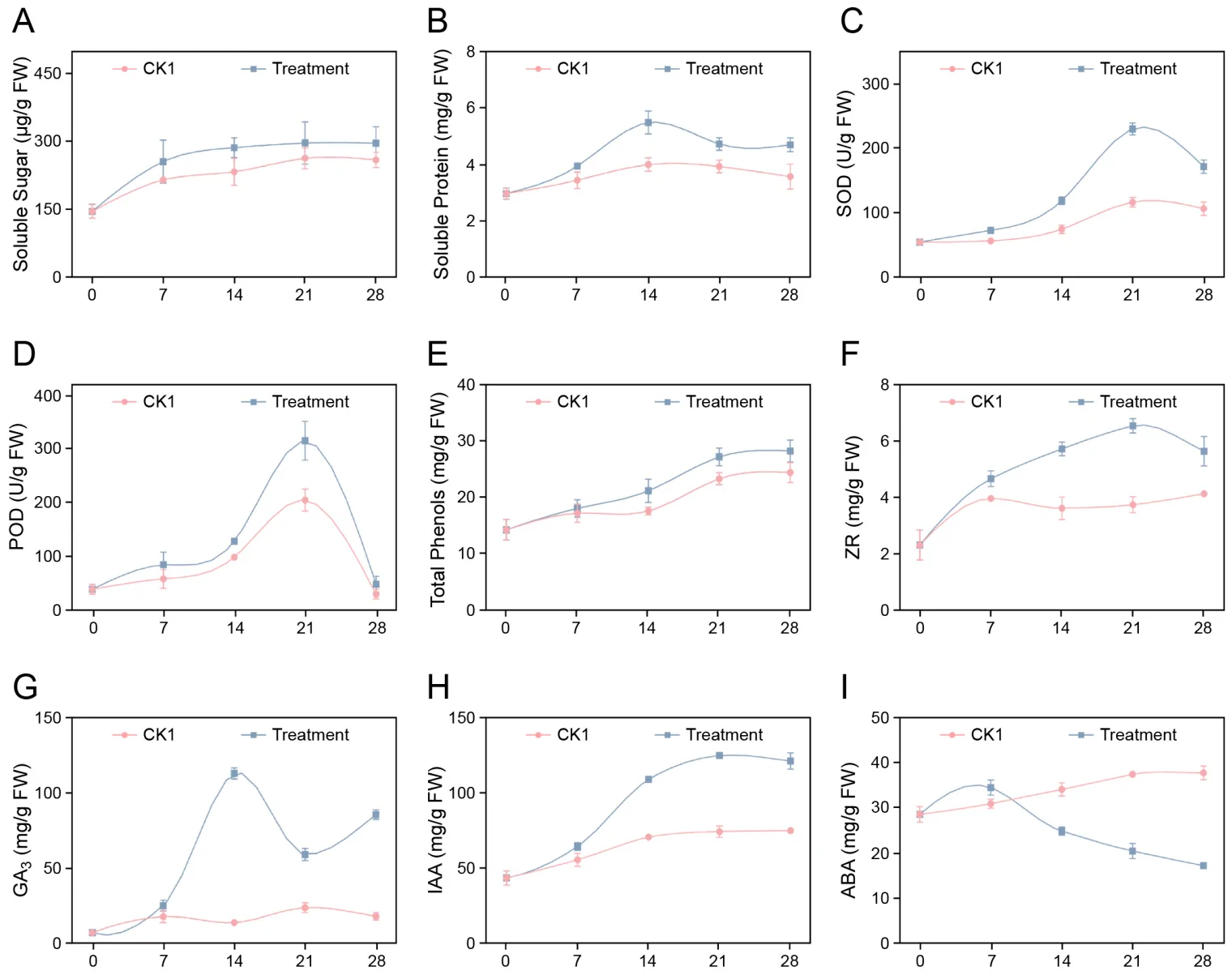
Dynamics of physiological parameters and endogenous hormones during shoot proliferation in *Camphora officinarum*. Note: (**A**) Soluble sugars; (**B**) soluble proteins; (**C**) SOD; (**D**) POD; (**E**) total phenolics; (**F**) ZR; (**G**) GA_3_; (**H**) IAA; and (**I**) ABA. Treatment: MS supplemented with 2.00 mg/L 6-BA, 0.20 mg/L NAA, and 0.05 mg/L KT. CK1: control on MS without plant growth regulators. Samples were collected on days 0, 7, 14, 21, and 28. ZR, GA_3_, IAA, and ABA contents are expressed as mg/g FW. Data are presented as the mean ± standard deviation (SD) of three biological replicates. FW, fresh weight.

During shoot proliferation, soluble sugar content increased rapidly over the first 7 days, after which the increase slowed. Treated plantlets generally had higher soluble sugar contents than the controls from days 7 to 28. Soluble protein content in treated plantlets increased from day 0 to day 14, peaked on day 14, and subsequently declined before remaining relatively stable. The control also reached a high soluble protein content on day 14, but its variation was smaller than that of the treatment (Figure 2A,B). The two parameters thus followed different temporal patterns: soluble sugar content remained high later in culture, whereas soluble protein content declined after its peak.

SOD and POD levels initially increased and then declined, peaking on day 21 in treated plantlets and decreasing by day 28. POD showed a particularly large decrease between days 21 and 28. Both SOD and POD levels were higher in the treatment than in the control at most sampling times (Figure 2C,D). Total phenolic content generally increased with culture duration in both groups. It was higher in treated plantlets later in culture, with the increase slowing between days 21 and 28 (Figure 2E).

Endogenous hormones exhibited distinct temporal patterns. In treated plantlets, ZR content increased from day 0 to day 21 and declined on day 28. IAA content increased rapidly between days 7 and 14, reached a high level on day 21, and decreased slightly on day 28. GA_3_ content peaked on day 14, declined on day 21, and increased again on day 28. ABA content in treated plantlets increased on day 7 and then declined continuously, whereas the control maintained relatively high ABA contents later in culture (Figure 2F–I). Overall, during the middle and later culture periods, treated plantlets had higher ZR, GA_3_, and IAA contents and lower ABA contents than the controls.

### 2.3. Differences in Shoot Physiological Parameters and Endogenous Hormones Among Plant Growth Regulator Formulations

**Figure 3:**
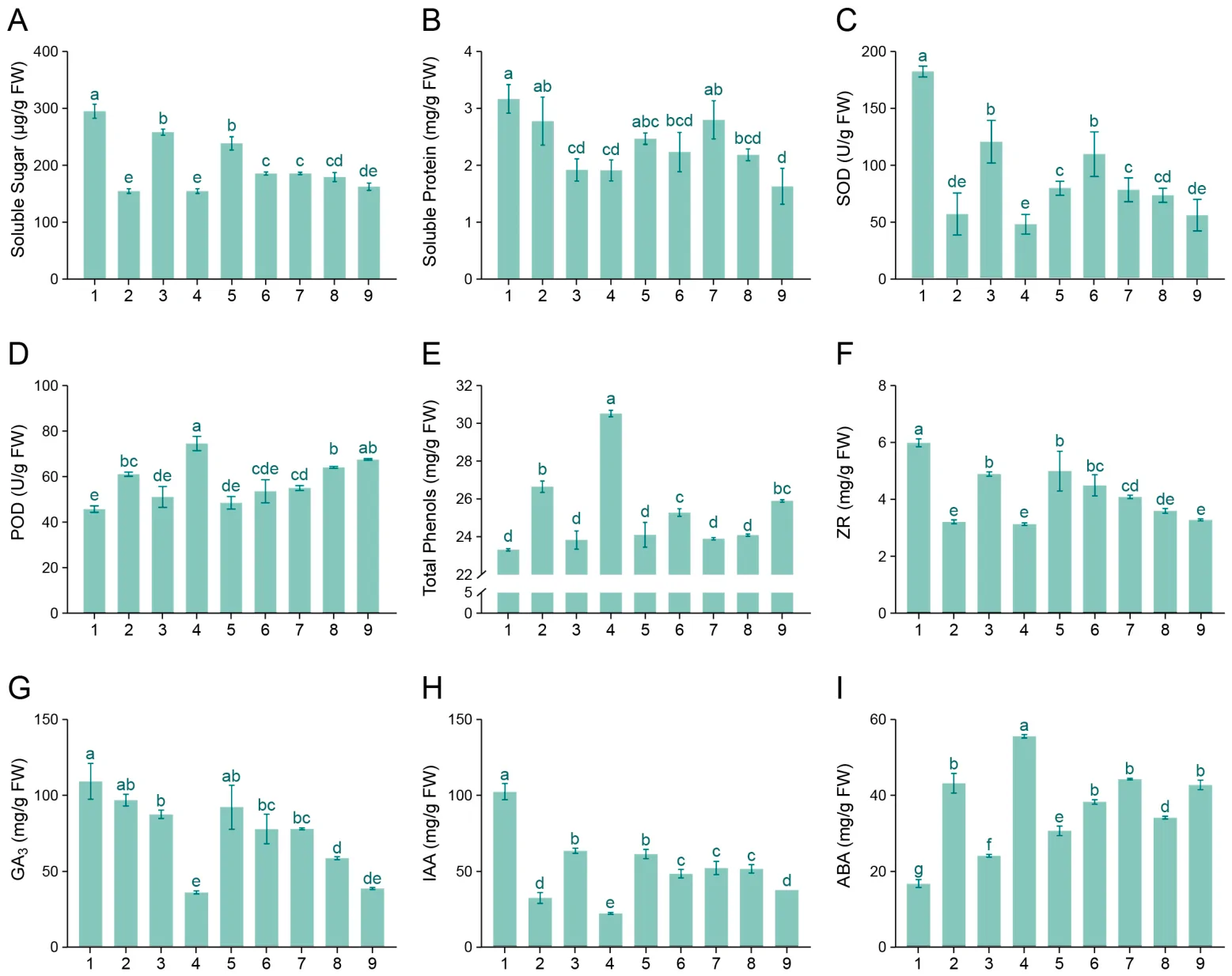
Differences in physiological parameters and endogenous hormones of *Camphora officinarum* shoots among plant growth regulator formulations. Note: (**A**) Soluble sugars; (**B**) soluble proteins; (**C**) SOD; (**D**) POD; (**E**) total phenolics; (**F**) ZR; (**G**) GA_3_; (**H**) IAA; and (**I**) ABA. Numbers 1–9 on the *x*-axis correspond to treatments J1–J9, respectively; their formulations are listed in Table 3. Samples were collected on day 28. ZR, GA_3_, IAA, and ABA contents are expressed as mg/g FW. Data are presented as the mean ± standard deviation (SD) of three replicates. For each parameter, treatments that do not share a lowercase letter differ significantly according to Tukey’s test (*p* < 0.05).

Shoot physiological parameters differed among formulations. Soluble sugar content was highest in J1, followed by J3 and J5, and was relatively low in J2 and J4. Soluble protein content was numerically highest in J1, although it did not differ significantly from J2 or J7; J9 had the lowest content (Figure 3A,B). Thus, the high proliferation coefficient in J1 coincided with relatively high soluble sugar and soluble protein contents, although the other treatments were not ranked identically for these parameters.

J1 had a higher SOD level than the other treatments, with J3 and J6 also showing relatively high levels. POD was highest in J4 and relatively low in J1 and J5. Total phenolic content was highest in J4 and relatively low in J1, J3, J5, J7, and J8, with no significant differences among the latter five treatments (Figure 3C–E). The high total phenolic content in J4 coincided with poor proliferation and growth, as also observed in the shoot photographs (Figure 1E).

Among the endogenous hormones, ZR and IAA contents were higher in J1 than in the other treatments. GA_3_ content was also numerically highest in J1, although it did not differ significantly from some other treatments. J4 had relatively low ZR, GA_3_, and IAA contents and the highest ABA content, whereas J1 had the lowest ABA content (Figure 3F–I). Overall, the favorable proliferation performance of J1 coincided with relatively high ZR and IAA contents and low ABA content.

### 2.4. Effects of Basal Media and Auxins on Adventitious Root Formation

With all treatments supplemented with 0.50 mg/L IBA, the control lacking basal nutrients (CK1) had a rooting rate of 51.11%, a mean root number of 0.83, and a mean root length of 7.33 mm. The corresponding values were 92.96%, 4.37, and 93.09 mm for 1/2MS; 75.56%, 1.74, and 47.32 mm for MS; and 82.22%, 2.98, and 82.62 mm for WPM. All three basal media produced significantly higher rooting rates and greater mean root lengths than CK. Mean root number was significantly higher on WPM and 1/2MS than in CK, whereas the difference between MS and CK was not significant. Among the three basal media, 1/2MS produced a significantly higher mean root number than MS or WPM. Rooting rate and mean root length were also significantly higher on 1/2MS than on MS, but neither differed significantly between 1/2MS and WPM (Table 2). Considering all three parameters, 1/2MS was selected as the basal medium for subsequent auxin screening.

**Figure 4:**
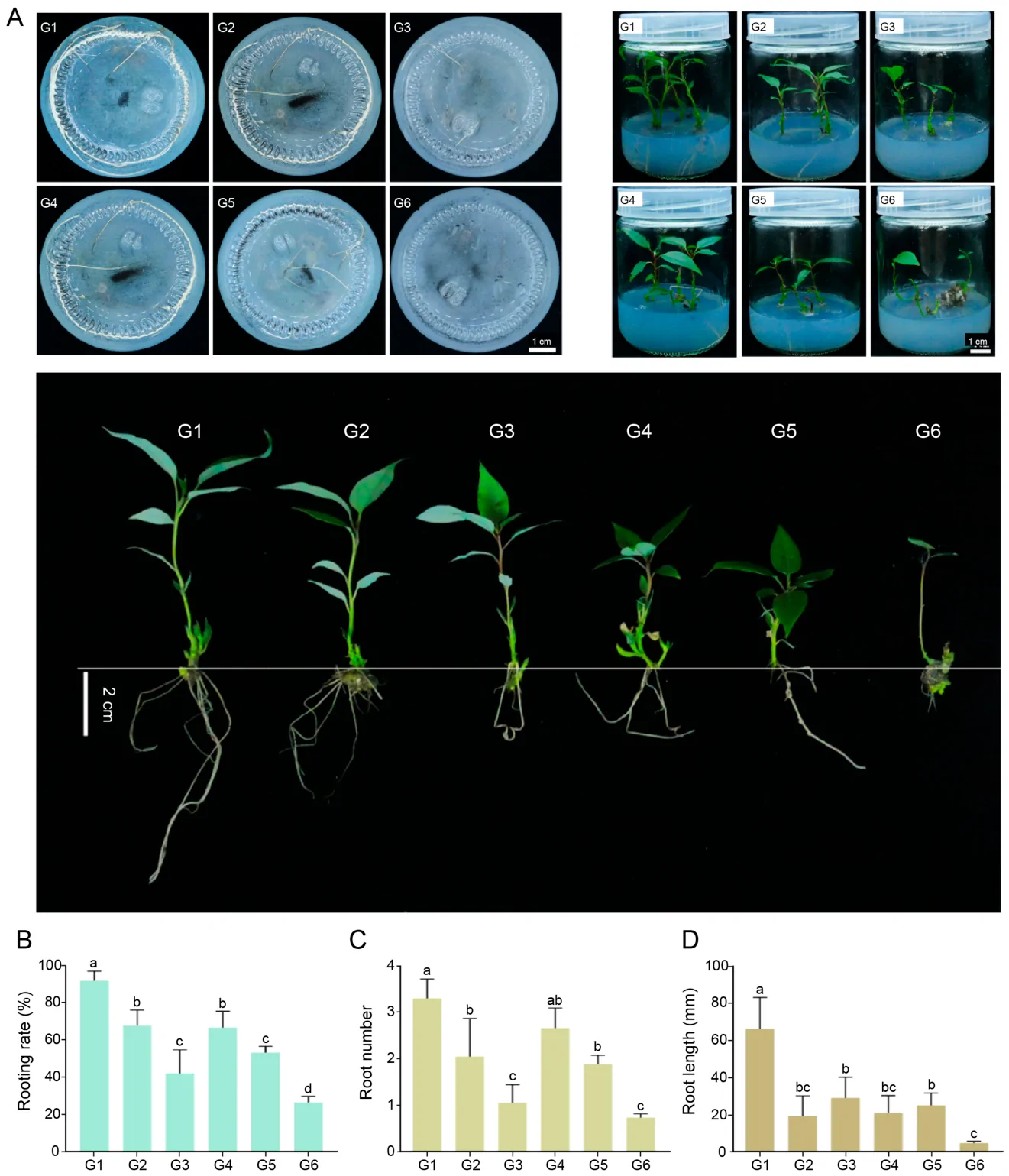
Effects of auxin treatments on adventitious root formation and root growth in *Camphora officinarum*. Note: (**A**) Rooting viewed from the bottoms of culture vessels (upper left), shoot growth within culture vessels (upper right), and whole-plantlet and root morphology (bottom) under treatments G1–G6. (**B**) Rooting rate; (**C**) mean root number; and (**D**) mean root length. Formulations G1–G6 are listed in Table 4. Rooting performance was assessed after 45 days of culture. Data in the graphs are presented as the mean ± standard deviation (SD) of three replicates. For each parameter, treatments that do not share a lowercase letter differ significantly according to Tukey’s test (*p* < 0.05). Scale bars in panel (**A**) represent 1 cm in the upper-left and upper-right photographs and 2 cm in the bottom photograph.

In vitro rooting, shoot growth, and root morphology under the different auxin treatments on 1/2MS are shown in Figure 4A. G1 (0.50 mg/L IBA) produced the highest rooting rate (92.22%), with a mean root number of 3.29 and a mean root length of 65.89 mm (Figure 4B–D). As IBA concentration increased from 0.50 to 1.00 and 2.00 mg/L, rooting rate decreased to 67.78% and 42.22%, respectively, and mean root number also declined. Mean root length decreased at 1.00 mg/L and increased again at 2.00 mg/L, but remained below that at 0.50 mg/L.

Under IAA treatment, rooting rate decreased with increasing concentration, reaching 66.67%, 53.33%, and 26.67% at 0.50, 1.00, and 2.00 mg/L, respectively (Figure 4B). At each concentration, IBA produced a higher rooting rate than IAA, but this advantage was not consistently observed for mean root number or mean root length (Figure 4C,D). Based on rooting rate and root growth, 1/2MS supplemented with 0.50 mg/L IBA was selected for the rooting time-course experiment.

Rooted plantlets were transplanted after acclimatization. Among the substrates tested, the 1:1 peat–perlite mixture produced the highest survival rate (67.78%), exceeding those of the other substrates (Table S4).

### 2.5. Dynamics of Physiological Parameters and Endogenous Hormones During Adventitious Root Formation

**Figure 5:**
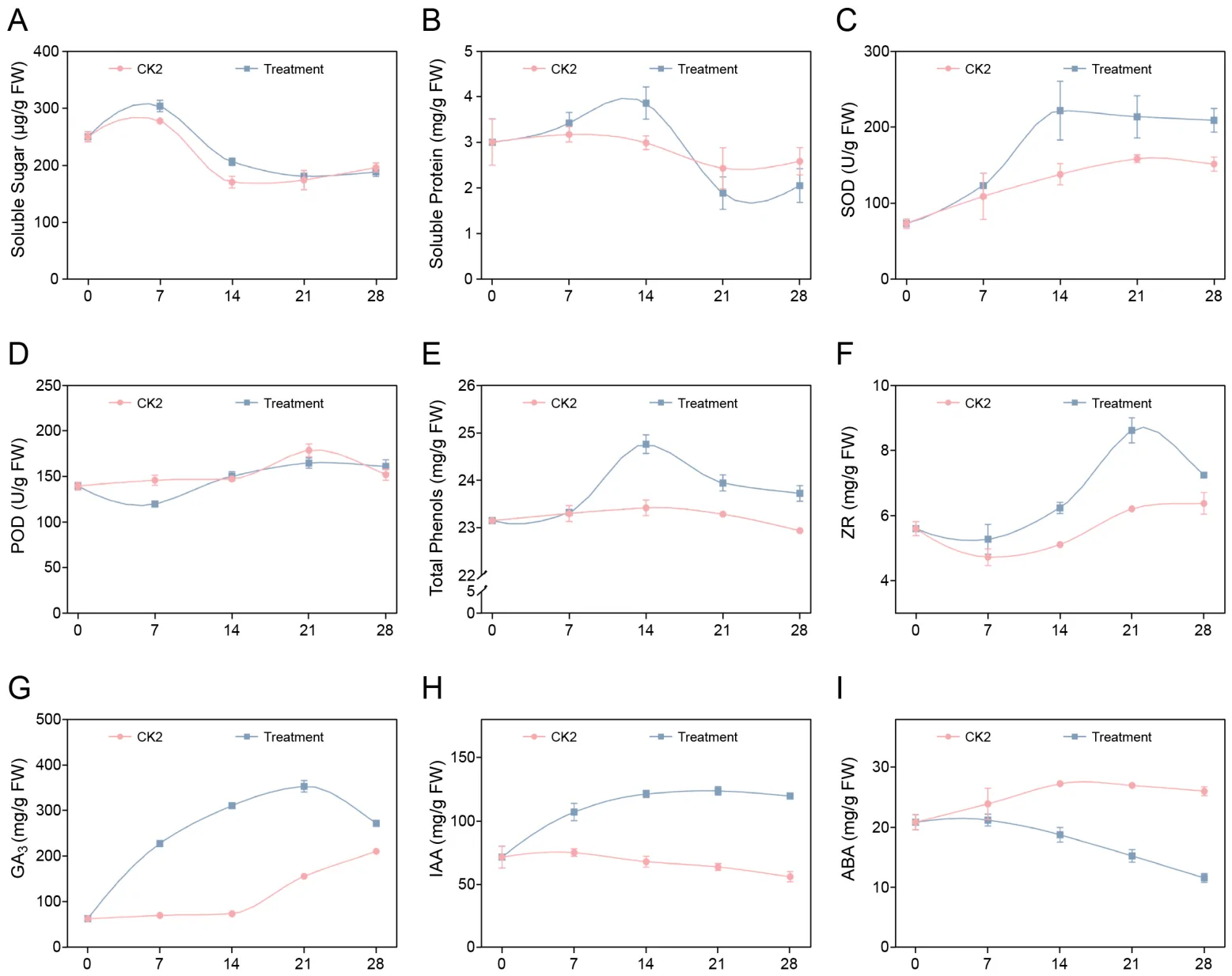
Dynamics of physiological parameters and endogenous hormones during adventitious root formation in *Camphora officinarum*. Note: (**A**) Soluble sugars; (**B**) soluble proteins; (**C**) SOD; (**D**) POD; (**E**) total phenolics; (**F**) ZR; (**G**) GA_3_; (**H**) IAA; and (**I**) ABA. Treatment: 1/2MS supplemented with 0.50 mg/L IBA. CK2: control on 1/2MS without auxins. Samples were collected on days 0, 7, 14, 21, and 28. ZR, GA_3_, IAA, and ABA contents are expressed as mg/g FW. Data are presented as the mean ± standard deviation (SD) of three replicates.

During rooting, soluble sugar content increased over the first 7 days, declined between days 7 and 21, and increased slightly on day 28. Treated plantlets had higher soluble sugar contents than the controls during the early and middle culture periods, but values were similar between the groups on days 21–28. Soluble protein content in treated plantlets peaked on day 14, declined markedly on day 21, and increased on day 28. The control showed smaller fluctuations, and soluble protein content was lower in the treatment later in culture (Figure 5A,B). In contrast to the general increase in soluble sugar content during shoot proliferation, a pronounced decline occurred during the middle period of rooting culture.

SOD levels in treated plantlets increased from day 0 to day 14, declined slightly thereafter, and remained relatively high. They were generally higher than in the controls during the middle and later culture periods. POD levels in treated plantlets decreased on day 7, subsequently increased to a high level on day 21, and declined slightly on day 28. Whether POD levels were higher in the treatment or the control depended on the sampling time (Figure 5C,D). Total phenolic content in treated plantlets peaked on day 14 and declined during days 21–28, whereas the control showed relatively small changes overall (Figure 5E).

ZR content initially decreased and then increased in both groups; in treated plantlets, it peaked on day 21 and declined on day 28. GA_3_ content in treated plantlets increased continuously from day 0 to day 21 and declined on day 28. IAA content increased early in culture and remained high during days 14–28. ABA content generally decreased in treated plantlets, whereas it increased in the control before day 14 and changed little thereafter (Figure 5F–I). Compared with the control, IBA-treated plantlets had higher ZR, GA_3_, and IAA contents and lower ABA contents during the middle and later culture periods.

### 2.6. Differences in Plantlet Physiological Parameters and Endogenous Hormones Among Auxin Treatments

**Figure 6:**
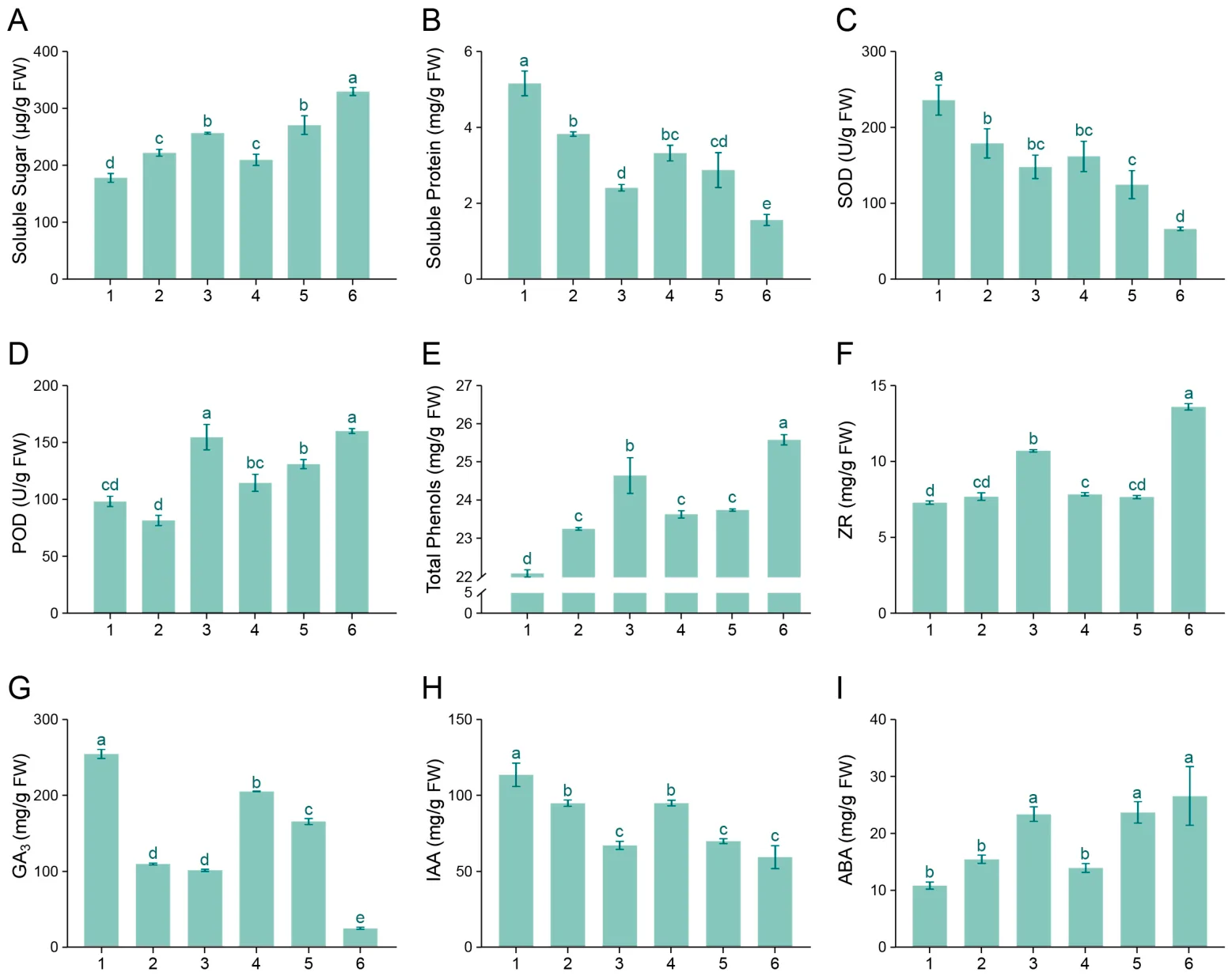
Differences in physiological parameters and endogenous hormones of *Camphora officinarum* plantlets among auxin treatments. Note: (**A**) Soluble sugars; (**B**) soluble proteins; (**C**) SOD; (**D**) POD; (**E**) total phenolics; (**F**) ZR; (**G**) GA_3_; (**H**) IAA; and (**I**) ABA. Numbers 1–6 on the *x*-axis correspond to treatments G1–G6, respectively; their formulations are listed in Table 4. Samples were collected on day 28. ZR, GA_3_, IAA, and ABA contents are expressed as mg/g FW. Data are presented as the mean ± standard deviation (SD) of three replicates. For each parameter, treatments that do not share a lowercase letter differ significantly according to Tukey’s test (*p* < 0.05).

Soluble sugar content increased with auxin concentration within both the IBA and IAA series, with the lowest content in G1 and the highest in G6. Soluble protein content showed the opposite overall pattern: it was highest in G1 and lowest in G6 and decreased with increasing concentration within each auxin series (Figure 6A,B). Thus, in this comparison of formulations, favorable rooting under the low-concentration IBA treatment coincided with low soluble sugar and high soluble protein contents.

SOD levels generally decreased with increasing auxin concentration within both the IBA and IAA series, with the highest level in G1 and the lowest in G6. POD levels were lowest in G2 and relatively high in G3 and G6. Total phenolic content was lowest in G1 and highest in G6 and generally increased with concentration in both auxin series (Figure 6C–E). These parameters differed in their responses to auxin type and concentration; in particular, treatment rankings for POD did not match those for SOD.

Among the endogenous hormones, ZR content was numerically lowest in G1 and highest in G6, with a relatively high value also observed in G3. GA_3_ and IAA contents were highest in G1. GA_3_ content was lowest in G6, and IAA content was relatively low in G3, G5, and G6. ABA content was relatively low in G1 and high in G3, G5, and G6 (Figure 6F–I). Overall, G1 had relatively high GA_3_ and IAA contents and low ZR and ABA contents, contrasting with the high ZR content associated with the formulation favorable for shoot proliferation.

### 2.7. Correlations of Physiological Parameters and Endogenous Hormones with Shoot Proliferation and Rooting Performance

**Figure 7:**
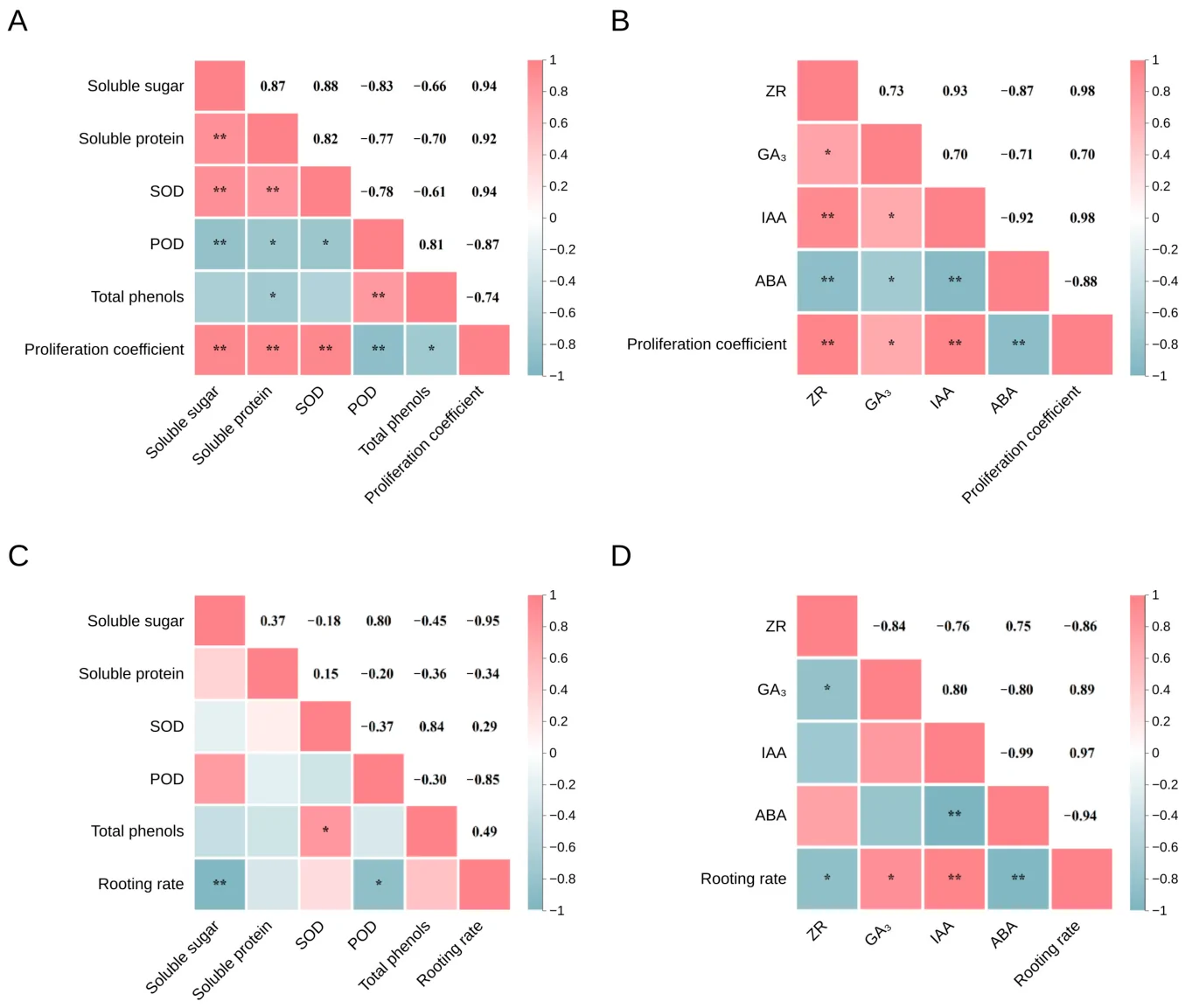
Correlations of physiological parameters and endogenous hormones with culture performance during shoot proliferation and rooting in *Camphora officinarum*. Note: (**A**,**B**) Correlations involving physiological parameters and endogenous hormones, respectively, during shoot proliferation. (**C**,**D**) Corresponding correlations during rooting. Numbers indicate Pearson correlation coefficients; red denotes positive correlations and blue denotes negative correlations. * *p* < 0.05; ** *p* < 0.01. Physiological parameters and hormone contents were measured on day 28; the proliferation coefficient and rooting rate were assessed on days 30 and 45, respectively.

Across formulations J1–J9, soluble sugar content, soluble protein content, and SOD levels measured on day 28 were significantly positively correlated with the proliferation coefficient measured on day 30, with correlation coefficients of 0.94, 0.92, and 0.94, respectively (all *p* ≤ 0.01). POD levels and total phenolic content were significantly negatively correlated with the proliferation coefficient, with coefficients of −0.87 (*p* ≤ 0.01) and −0.74 (*p* ≤ 0.05), respectively (Figure 7A). Among the endogenous hormones, ZR and IAA contents were significantly positively correlated with the proliferation coefficient (r = 0.98 for both, *p* ≤ 0.01). GA_3_ content was also significantly positively correlated with the proliferation coefficient (r = 0.70, *p* ≤ 0.05), whereas ABA content was significantly negatively correlated (r = −0.88, *p* ≤ 0.01; Figure 7B).

Across treatments G1–G6, soluble sugar content and POD levels measured on day 28 were significantly negatively correlated with rooting rate measured on day 45, with coefficients of −0.95 (*p* ≤ 0.01) and −0.85 (*p* ≤ 0.05), respectively. Soluble protein content, SOD levels, and total phenolic content were not significantly correlated with rooting rate (Figure 7C). Among the endogenous hormones, IAA and GA_3_ contents were significantly positively correlated with rooting rate, with coefficients of 0.97 (*p* ≤ 0.01) and 0.89 (*p* ≤ 0.05), respectively. ZR and ABA contents were significantly negatively correlated with rooting rate, with coefficients of −0.86 (*p* ≤ 0.05) and −0.94 (*p* ≤ 0.01), respectively (Figure 7D).

In the formulation comparisons at each stage, IAA and GA_3_ contents were positively correlated with the respective proliferation coefficient or rooting rate, whereas ABA content was negatively correlated with both. Soluble sugar and ZR contents were positively correlated with the proliferation coefficient but negatively correlated with rooting rate. These results describe associations between physiological parameters, endogenous hormone contents, and culture performance across formulations; they do not establish direct promotive or inhibitory effects on shoot proliferation or adventitious root formation.

## 3. Discussion

### 3.1. Differences in Culture Conditions for Shoot Proliferation and Adventitious Root Formation

MS was more suitable for shoot proliferation, whereas 1/2MS performed well during rooting, indicating that the tested material responded differently to basal media at the two stages. This differs from the rooting of embryo-derived *C. officinarum* shoots on hormone-free MS reported previously [3], illustrating why culture protocols should be evaluated in relation to their explant source and regeneration pathway. This study compared the overall effects of basal media without separately testing total salt concentration, nitrogen form, or individual ions. The rooting response to 1/2MS therefore cannot be attributed to a particular nutrient component.

The favorable proliferation response to 6-BA, NAA, and KT supports evaluating regulator combinations together with their concentration ranges. Effects of plant growth regulator combinations on proliferation and growth have also been reported in *G. jasminoides*, *Broussonetia papyrifera*, and blueberry [5,9,10]. For example, a combination of 6-BA, IAA, and KT supported adventitious bud regeneration in *G. jasminoides* [5], whereas NAA was included in the selected formulation here. The different auxins and regeneration systems limit direct transfer of either formulation. Moreover, proliferation and plantlet height did not show identical responses to the tested formulations. Medium selection should therefore consider both the number of newly formed usable shoots and shoot growth. These results support the combined use of 6-BA, NAA, and KT for the tested material but do not demonstrate a specific synergistic mechanism among the three regulators.

During rooting, 0.50 mg/L IBA produced the highest rooting rate among the concentrations tested, and further increases in concentration reduced rooting. In the citral chemotype of *Cinnamomum bodinieri*, the highest rooting response occurred at 1.50 mg/L IBA after 25 days [4], compared with the assessment after 45 days here. Differences in plant material, culture conditions, and evaluation time prevent a direct ranking of the two protocols. Combining rooting rate, mean root number, and mean root length provides a more complete assessment of culture performance. Because mean root number here included unrooted plantlets, it reflects both rooting frequency and root production and should not be interpreted as root number per rooted plantlet.

Despite an in vitro rooting rate of 92.22% under the selected auxin treatment, the highest survival rate recorded 30 days after transplanting was only 67.78%, obtained with the peat–perlite substrate. Although these percentages refer to different assessment populations, the limited post-transfer survival identifies establishment after transplanting as a bottleneck in practical propagation. Rooting performance alone is therefore insufficient to assess the practical effectiveness of this culture system. Optimization of acclimatization should be pursued as a distinct objective, with post-transfer survival and subsequent plant growth as key evaluation criteria. Future experiments should separately assess the duration of vessel ventilation, gradual reduction in humidity, and post-transfer light and water management. These factors were not independently evaluated here, and their contributions to transplant mortality remain unresolved.

### 3.2. Physiological Changes During Shoot Proliferation and Rooting

The contrasting associations of soluble sugar content with proliferation and rooting were more informative than the individual temporal peaks: sugar content was positively associated with the proliferation coefficient across shoot formulations but negatively associated with rooting rate across auxin treatments. In *Cinnamomum bodinieri*, IBA-induced rooting was accompanied by changes in carbohydrate-metabolism gene expression in the micro-shoot base [4]. This provides a plausible metabolic context for the present observations, although local transcriptional responses and whole-plantlet sugar contents represent different levels of evidence. Decreasing sugar content may reflect substrate utilization during rooting, but may also be influenced by changes in biomass and the tissue composition of samples. The present measurements do not distinguish uptake, synthesis, utilization, or growth dilution; consequently, neither high sugar content during proliferation nor low sugar content during rooting establishes a causal contribution to performance.

Soluble protein content was positively associated with proliferation but was not significantly associated with rooting rate. Its relationship with growth therefore also depended on the culture stage and comparison being made. Total soluble protein measurements do not identify the proteins involved, so these results cannot be assigned to increased synthesis of particular growth-related enzymes.

SOD and POD showed different relationships with culture performance: the positive SOD association was restricted to proliferation, whereas POD was negatively associated with the performance measure at both stages. In *Acer mono* cuttings, rejuvenation altered hormone dynamics and reduced POD activity [7], while comparisons of *Eucommia ulmoides* varieties revealed differing physiological trajectories during rooting [8]. These studies show that antioxidant-related responses depend on material and treatment context, rather than supporting a universal relationship between a higher enzyme value and better rooting. Without measurements of reactive oxygen species and relevant functional assays, changes in SOD and POD levels alone also cannot establish the extent of oxidative damage or reactive oxygen species-scavenging capacity.

The negative association between total phenolics and proliferation, together with poor growth in the high-phenolic treatment, is compatible with a contribution of phenolic accumulation to unfavorable culture conditions. In *Artemisia annua*, inhibiting phenylalanine ammonia-lyase reduced phenolic accumulation and browning and improved growth on cytokinin-containing medium [11]. A study of date palm cultures also linked reduced browning with changes in total phenols and peroxidase activity [12]. These studies provide context for the association observed here, but the present study did not manipulate phenolic biosynthesis. However, total phenolic content was not significantly correlated with rooting rate across rooting treatments, indicating that this relationship cannot be directly extended to the rooting stage. The aggregate assay also cannot identify which phenolic compounds or oxidation products might be involved.

### 3.3. Endogenous Hormonal Changes and Their Relationships with Shoot Proliferation and Rooting

Higher IAA and lower ABA contents were associated with favorable culture performance at both stages, whereas the association of ZR differed between proliferation and rooting. These shared and contrasting features offer a more useful interpretation than assigning each hormone a fixed promotive or inhibitory role. Studies of *G. jasminoides* tissue culture and persimmon rooting likewise examined endogenous hormonal profiles alongside responses to exogenous regulators [5,6], supporting the relevance of hormonal status to propagation while leaving its interpretation dependent on the culture system.

For IBA-treated plantlets, the positive relationship between IAA content and rooting is consistent with the involvement of endogenous auxin status in adventitious root formation. The expression changes in auxin-related genes reported for *Cinnamomum bodinieri* provide additional evidence of auxin-associated responses during rooting in a related species [4]. However, our measurements cannot determine whether the observed IAA profile arose from IBA conversion, endogenous synthesis, transport, or degradation. These are possible explanations, not mechanisms demonstrated in this experiment. GA_3_ content may also reflect overall plantlet growth, so its positive correlation with rooting rate does not establish a direct role in promoting root induction.

ZR showed opposite correlations in the formulation comparisons at the two stages: a positive correlation with the proliferation coefficient and a negative correlation with rooting rate. Its increase during part of the rooting time course does not contradict this pattern: temporal changes under one formulation and differences among formulations at a common sampling time answer different questions. Whole-plantlet ZR measurements also cannot resolve cytokinin status at the root initiation site. Thus, the results identify a stage-associated correlation pattern, rather than demonstrate that ZR controls the transition between proliferation and rooting.

The ABA association also requires consideration of the regeneration pathway. In *C. officinarum* somatic embryogenesis, ABA/IAA and ABA/GA_3_ ratios were highest in somatic embryos compared with the callus materials examined [13]. That result differs in its comparison and developmental context from the negative associations between ABA content and culture performance observed here. Hormone ratios in embryogenic tissues and individual hormone contents in whole plantlets are not interchangeable measures. Together, these findings argue against interpreting low ABA as a general requirement for all forms of regeneration.

### 3.4. Stage-Dependent Responses and Study Limitations

The contribution of this study is the combined assessment of propagation performance, temporal responses, and associations across formulations in the tested donor material. The contrasting sugar and ZR associations indicate that an indicator associated with favorable proliferation need not have the same relationship with rooting. Time-course and formulation comparisons provide complementary information, but this study did not test whether the measured parameters can predict proliferation or rooting in other plant materials or under other culture conditions.

Physiological and hormonal measurements were performed on whole plantlets and thus represent their integrated status. The relative proportions of different tissues change as new shoots or roots form, and some differences in measured contents may arise from changes in tissue composition. In addition, the two stages used different basal media and exogenous regulators, so the observed differences reflect the combined effects of culture conditions and development, which could not be separated here. Tissue-specific measurements and targeted perturbation of the proposed metabolic or hormonal processes would be needed to distinguish local responses and test causal explanations.

In the formulation experiments, physiological parameters and hormone contents were measured on day 28, whereas proliferation and rooting were assessed on days 30 and 45, respectively. The correlations therefore describe associations between earlier physiological status and subsequent culture performance across treatments; they do not represent longitudinal measurements of the same individual plantlets or establish causality. The conclusions are limited to the donor tree and conditions tested and require validation in other individuals or genotypes. Furthermore, essential oil composition and genetic fidelity were not assessed in regenerated plantlets, so the retention of the donor tree’s essential oil traits in its propagated progeny remains unknown. Future work should assess the essential oil profiles, including essential oil composition and relative citral content, and genetic fidelity of regenerated plants to evaluate the retention of the donor tree’s characteristics.

## 4. Materials and Methods

### 4.1. Plant Material and Culture Conditions

Plant material was collected from a *C. officinarum* nursery in Huangsha Village, Xianshui Town, Quanzhou County, Guilin, Guangxi, China (25.81834° N, 110.81008° E). The donor tree was vigorous and showed no visible signs of pests or disease. Leaf essential oil was extracted by hydrodistillation using a modified Clevenger apparatus [1], and its composition was analyzed by gas chromatography (GC) [2]. The leaf essential oil content of the donor tree was 0.86%, and the relative citral content was 88%. Young current-year branches were collected and transported to the laboratory under cool, moist conditions. Leaves were removed, and bud-bearing stem segments were excised as explants. All media contained 30 g/L sucrose and 6 g/L agar, were adjusted to pH 5.4–5.8, and were autoclaved at 121 °C and 0.1 MPa for 20 min. Cultures were maintained at 22–24 °C under a 12 h light/12 h dark photoperiod and an illuminance of 2000–2400 lx.

### 4.2. Establishment of Aseptic Cultures and Axillary Bud Initiation

Bud-bearing stem segments were immersed in water containing a small amount of detergent, and surface debris was removed with a soft brush. After soaking for 20 min, the material was rinsed under running tap water for 2–3 h. It was then transferred to a laminar flow cabinet, rinsed twice with sterile water, and cut into 1–2 cm bud-bearing segments.

Surface disinfection conditions were screened in two steps. First, explants were treated with 75% ethanol for 30 s, followed by either a 1:1000 dilution of carbendazim for 30 min, a 20% (*v*/*v*) dilution of commercial sodium hypochlorite solution for 10 min, or 0.1% HgCl_2_ for 7 min, to compare the effectiveness of the disinfectant combinations. Subsequently, the ethanol treatment was fixed at 30 s, and treatment with 0.1% HgCl_2_ for 3, 5, 7, or 10 min was tested to determine a suitable exposure time. Disinfected explants were rinsed three times with sterile water, damaged tissue was removed, and surface moisture was blotted off with sterile filter paper before inoculation onto Murashige and Skoog (MS) medium. Each treatment comprised three replicates of 30 culture vessels, with one explant per vessel, giving 90 explants per treatment. Survival, contamination, browning, and bud break rates were recorded after 15 days of culture.

Axillary bud initiation was examined on MS basal medium using single-factor experiments to screen 6-benzyladenine (6-BA) and indole-3-butyric acid (IBA) concentrations. Initially, IBA was fixed at 0.50 mg/L, and 6-BA was supplied at 0.50, 1.00, 2.00, 3.00, or 4.00 mg/L. Based on these results, 6-BA was fixed at 2.00 mg/L, and IBA was supplied at 0.05, 0.10, 0.50, or 1.00 mg/L. Each treatment comprised three replicates of 30 vessels, with one explant per vessel, giving 90 explants per treatment. Survival, browning, and bud break rates were recorded after 30 days to identify a suitable medium for axillary bud initiation.

### 4.3. Screening and Optimization of Shoot Proliferation Conditions

Vigorous aseptic shoots of uniform growth were trimmed to approximately 1.5 cm and transferred to fresh medium for subculture. Medium screening comprised three stages: comparison of basal media, screening of plant growth regulator combinations, and optimization of regulator concentrations.

First, MS, modified MS, and woody plant medium (WPM) were compared for their effects on shoot proliferation. Modified MS consisted of MS medium supplemented with an additional 300 mg/L Ca(NO_3_)_2_·4H_2_O. All three media contained 2.00 mg/L 6-BA, 0.20 mg/L 1-naphthaleneacetic acid (NAA), and 0.05 mg/L kinetin (KT).

Based on the basal medium screening, five plant growth regulator combinations were tested on MS medium: Z1, 1.00 mg/L 6-BA + 0.10 mg/L IBA; Z2, 1.00 mg/L 6-BA + 0.10 mg/L NAA; Z3, 1.00 mg/L 6-BA + 0.10 mg/L NAA + 0.10 mg/L KT; Z4, 1.00 mg/L 6-BA + 0.10 mg/L NAA + 0.10 mg/L zeatin (ZT); and Z5, 1.00 mg/L 6-BA + 0.10 mg/L NAA + 0.10 mg/L gibberellic acid (GA_3_). Their effects on shoot proliferation were compared.

The concentrations of 6-BA, NAA, and KT were subsequently optimized using an L_9_(3^3^) orthogonal design (Table 3). Three concentrations were tested for each factor: 0.50, 1.00, and 2.00 mg/L for 6-BA; 0.05, 0.10, and 0.20 mg/L for NAA; and 0.05, 0.10, and 0.20 mg/L for KT.

Each treatment comprised three replicate groups established before the experiment, with 30 culture vessels per group and three aseptic shoots per vessel. Observations were recorded and summarized separately for each group to calculate the proliferation coefficient and mean plantlet height. After 30 days of culture, the number of usable shoots and plantlet height were measured, and shoot growth and browning were recorded. Shoots taller than 1 cm were considered usable. In the plant growth regulator combination screening experiment, the number of newly formed usable shoots was calculated by subtracting the initial number of inoculated shoots. The proliferation coefficient was calculated as (total number of usable shoots after culture − initial number of inoculated shoots)/initial number of inoculated shoots. Suitable basal media, plant growth regulator combinations, and concentrations were selected based on the proliferation coefficient and plantlet height.

**Table 3:** L_9_(3^3^) orthogonal design for shoot proliferation in *Camphora officinarum*.

Treatment	6-BA (mg/L)	NAA (mg/L)	KT (mg/L)
J1	2.00	0.20	0.05
J2	0.50	0.10	0.20
J3	2.00	0.05	0.20
J4	0.50	0.20	0.10
J5	1.00	0.20	0.20
J6	2.00	0.10	0.10
J7	1.00	0.10	0.05
J8	1.00	0.05	0.10
J9	0.50	0.05	0.05

Note: All treatments used MS as the basal medium.

### 4.4. Experimental and Sampling Design for the Shoot Proliferation Stage

The treatment medium was MS supplemented with 2.00 mg/L 6-BA, 0.20 mg/L NAA, and 0.05 mg/L KT, and the control was MS without plant growth regulators. Whole aseptic plantlets were collected on days 0, 7, 14, 21, and 28 to determine soluble sugar, soluble protein, and total phenolic contents; SOD and POD levels; and zeatin riboside (ZR), GA_3_, indole-3-acetic acid (IAA), and abscisic acid (ABA) contents, thereby characterizing physiological and endogenous hormonal dynamics during shoot proliferation. On day 0, the treatment and control groups were sampled separately to provide their respective baseline samples.

To compare physiological status among plant growth regulator concentration combinations, whole plantlets from treatments J1–J9 were collected on day 28 for the same measurements. Correlations were analyzed between these parameters and the proliferation coefficient recorded on day 30 for the corresponding treatments. The time-course experiment included three biological replicates for each treatment at each sampling time, and the formulation comparison included three biological replicates per treatment. Each replicate consisted of a pooled sample of 10 plantlets with uniform growth. Samples were immediately frozen in liquid nitrogen and stored at −80 °C until analysis.

### 4.5. Adventitious Root Induction and Sampling Design

Vigorous, uniformly growing shoots from subcultures were cut into approximately 1.5 cm stem segments for rooting. First, rooting was compared among MS, half-strength MS (1/2MS), WPM, and a control without a basal nutrient formulation (CK1). All four treatments contained 0.50 mg/L IBA, 30 g/L sucrose, and 6 g/L agar. Based on these results, 1/2MS was selected as the basal medium and supplemented with either IBA or indole-3-acetic acid (IAA) at 0.50, 1.00, or 2.00 mg/L to compare the effects of auxin type and concentration on adventitious root induction (Table 4). Each treatment comprised three replicate groups established before the experiment, with 30 culture vessels per group and three shoots per vessel. After 45 days of culture, the number of rooted plantlets, root number, and root length were recorded and summarized separately for each replicate group to calculate rooting rate, mean root number, and mean root length.

Observations were recorded and summarized separately for each group to calculate rooting rate, mean root number, and mean root length. The number of rooted plantlets, root number, and root length were recorded after 45 days, and rooting rate, mean root number, and mean root length were calculated. Rooting rate was the percentage of inoculated plantlets that formed roots. Mean root number was calculated as the total number of roots on all assessed plantlets divided by the total number of assessed plantlets, with unrooted plantlets assigned a root count of zero. Mean root length was calculated as the sum of the lengths of measured roots divided by the number of roots measured.

For the rooting time-course experiment, the treatment medium was 1/2MS supplemented with 0.50 mg/L IBA, and the control was 1/2MS without auxins (CK2). Whole plantlets were collected on days 0, 7, 14, 21, and 28 to determine soluble sugar, soluble protein, and total phenolic contents; SOD and POD levels; and ZR, GA_3_, IAA, and ABA contents, thereby characterizing physiological and endogenous hormonal dynamics during adventitious root formation. Whole plantlets from treatments G1–G6 were also collected on day 28 for the same measurements, and correlations were analyzed between these parameters and the rooting rates recorded on day 45 for the corresponding treatments. Three biological replicates were collected for each treatment or sampling time, with each replicate consisting of a pooled sample of five plantlets with uniform growth. Samples were immediately frozen in liquid nitrogen and stored at −80 °C until analysis.

**Table 4:** Auxin treatments for adventitious root induction in *Camphora officinarum*.

Treatment	IBA (mg/L)	IAA (mg/L)
G1	0.50	—
G2	1.00	—
G3	2.00	—
G4	—	0.50
G5	—	1.00
G6	—	2.00

Note: All treatments used 1/2MS as the basal medium. “—” indicates that the auxin was not added.

### 4.6. Measurement of Physiological Parameters

Soluble sugar content was determined using the anthrone colorimetric method [14]. Frozen tissue (0.20 g) was homogenized in 2 mL of distilled water and extracted in a boiling water bath for 10 min. After cooling, the homogenate was centrifuged at 4000 rpm for 10 min. The supernatant was diluted as appropriate, and absorbance was measured at 620 nm. A calibration curve was constructed using glucose as the standard, and results were expressed on a fresh-weight basis.

Soluble protein content was determined using the Coomassie Brilliant Blue colorimetric method [15]. Frozen tissue (0.20 g) was homogenized on ice in 1.8 mL of 0.1 mol/L phosphate buffer (pH 7.0–7.4) and centrifuged at 12,000 rpm for 10 min at 4 °C. The supernatant was collected, and absorbance was measured at 595 nm. A calibration curve was constructed using bovine serum albumin as the standard, and results were expressed on a fresh-weight basis.

SOD and POD levels were determined using plant superoxide dismutase (SOD) and peroxidase (POD) ELISA kits, respectively (Shanghai Yingxin Laboratory Equipment Co., Ltd., Shanghai, China; catalog numbers TY61208-96T and TY98237-96T, respectively). Frozen tissue (0.20 g) was thoroughly ground in liquid nitrogen and homogenized in 1.8 mL of 0.1 mol/L phosphate buffer (pH 7.0–7.4). The homogenate was centrifuged under refrigerated conditions, and the supernatant was used for analysis. Assays were performed according to the manufacturers’ instructions. Absorbance was measured at 450 nm for SOD and 420 nm for POD, and their levels were calculated using the respective calibration curves.

Total phenolic content was determined using the Folin–Ciocalteu colorimetric method described in reference [16], with modifications. Frozen tissue (0.20 g) was ground in liquid nitrogen, mixed with 4 mL of methanol, and extracted in the dark at room temperature for 48 h. The extract was centrifuged at 10,000 rpm for 10 min. An aliquot of the supernatant (50 μL) was mixed with 750 μL of 10% Folin–Ciocalteu reagent and 135 μL of distilled water, followed by 600 μL of 700 mmol/L Na_2_CO_3_ solution. The mixture was mixed again and allowed to react at room temperature for 2 h. Absorbance was measured at 765 nm. A calibration curve was constructed using gallic acid as the standard, and results were expressed on a fresh-weight basis.

### 4.7. Determination of Endogenous Hormones

Zeatin riboside (ZR), GA_3_, IAA, and abscisic acid (ABA) contents were determined by high-performance liquid chromatography (HPLC) using a published method with modifications [17]. Frozen tissue (0.20 g) was homogenized in precooled 80% methanol, extracted by ultrasonication for 30 min, and centrifuged at 4000 rpm for 15 min at 4 °C. The residue was re-extracted three times with precooled 80% methanol. The supernatants were combined and concentrated under reduced pressure to remove methanol. Pigments were extracted with 2 mL of petroleum ether; this step was repeated three times, and the petroleum ether phase was discarded. The lower phase was further concentrated under reduced pressure and adjusted to pH 2.5–2.9 using 2.0 mol/L HCl. It was then extracted three times with an equal volume of ethyl acetate. The ethyl acetate fractions were combined and evaporated to dryness under nitrogen. The residue was dissolved in HPLC-grade methanol, brought to a final volume of 2 mL, and passed through a 0.22 μm organic-solvent-compatible membrane filter before analysis.

Chromatographic analysis was performed using an HPLC system equipped with an SPD-M20A diode-array detector (Shimadzu Corporation, Kyoto, Japan) and a Shim-pack Scepter C18-120 column (5 μm, 4.6 mm × 250 mm; Shimadzu Corporation, Kyoto, Japan) maintained at 35 °C. Mobile phase A was HPLC-grade methanol, and mobile phase B was an aqueous solution of glacial acetic acid at pH 3.2. Isocratic elution was performed at an A:B ratio of 45:55 (*v*/*v*), a flow rate of 1.0 mL/min, and an injection volume of 20 μL. ZR, IAA, and ABA were detected at 270 nm, and GA_3_ was detected at 206 nm. Hormones were identified by comparison with the retention times and UV absorption characteristics of authentic standards. Calibration curves were constructed with standard concentration (X, mg/L) on the *x*-axis and peak area (Y) on the *y*-axis. The regression equations for ZR, GA_3_, IAA, and ABA were Y = 27,564X − 74,612 (R^2^ = 0.9993), Y = 569.413X + 2823.80 (R^2^ = 0.9992), Y = 10,854.7X – 38,693.1 (R^2^ = 0.9994), and Y = 45,721.4X − 120,939 (R^2^ = 0.9991), respectively. Quantification was performed using the external standard method. All endogenous hormone contents were expressed as mg/g fresh weight (FW).

### 4.8. Acclimatization and Transplanting

After approximately 45 days of rooting culture, plantlets with intact root systems, fully expanded leaves, and uniform growth were selected and acclimatized in the culture room for 7 days with the vessel lids removed. Plantlets were removed from the vessels, and residual medium was washed from the roots. The roots were treated with 0.1% (*w*/*v*) carbendazim solution for 2 min before transplanting into one of six substrates: yellow soil; peat and yellow soil (1:1); nutrient soil and yellow soil (1:1); peat and perlite (1:1); nutrient soil and perlite (1:1); or vermiculite, perlite, and river sand (2:2:3). Ninety plantlets were transplanted into each substrate, with three replicates of 30 plantlets. The transplants were covered with plastic film for 10 days to retain moisture, and survival was assessed 30 days after transplanting. Survival rates in the different substrates are presented in Table S4, and the growth of plantlets transplanted into the peat–perlite mixture is shown in Figure 1F.

### 4.9. Statistical Analysis

Data were organized in Microsoft Excel (Microsoft Corporation, Redmond, WA, USA) and analyzed using IBM SPSS Statistics 27 (IBM Corp., Armonk, NY, USA). Graphs were prepared using GraphPad Prism 2020 (GraphPad Software, Boston, MA, USA). For comparisons of shoot proliferation and rooting performance among treatments, statistical analyses used one summary value from each of the three predefined replicate groups per treatment (*n* = 3). Each group-level value was calculated from observations across its 30 culture vessels according to the definitions of the respective indices. Individual shoots and culture vessels were not entered separately as replicates in these analyses. Treatment means and standard deviations were calculated from the three group-level values. Differences among basal media, plant growth regulator combinations, auxin treatments, and transplanting substrates were evaluated by one-way analysis of variance (ANOVA). Two-way ANOVA was used for the shoot proliferation and rooting time-course experiments to assess the effects of treatment, culture duration, and their interaction on physiological parameters and endogenous hormone contents. After confirming normality and homogeneity of variance, multiple comparisons were performed using Tukey’s test. Pearson correlation analysis was used to evaluate relationships between physiological parameters and endogenous hormone contents measured on day 28 and the proliferation coefficient or rooting rate. Data are presented as the mean ± standard deviation (SD), and differences were considered significant at *p* < 0.05. For the orthogonal experiment, range analysis was used to compare the effects of 6-BA, NAA, and KT on the proliferation coefficient, and ANOVA was used to test the significance of each factor. The proliferation coefficient was calculated as (total number of usable shoots after culture − initial number of inoculated shoots)/initial number of inoculated shoots. Rooting rate (%) was calculated as the number of rooted plantlets/total number of inoculated plantlets × 100. Mean root number was calculated as the total number of roots on all assessed plantlets/total number of assessed plantlets, with unrooted plantlets assigned a root count of zero. Mean root length was calculated as the sum of measured root lengths/total number of roots measured. Transplant survival (%) was calculated as the number of surviving transplants/total number of transplanted plantlets × 100.

## 5. Conclusions

Among the culture conditions tested, MS supplemented with 2.00 mg/L 6-BA, 0.20 mg/L NAA, and 0.05 mg/L KT was suitable for shoot proliferation of the citral-rich *C. officinarum* material, yielding a proliferation coefficient of 8.16. For adventitious root induction, 1/2MS supplemented with 0.50 mg/L IBA produced a rooting rate of 92.22%. Soluble sugar content generally increased during shoot proliferation but declined during the middle period of rooting culture. In the formulation comparisons at each stage, IAA content was positively correlated with the respective culture performance measure and ABA content was negatively correlated, whereas soluble sugar and ZR contents showed opposite correlations between stages. These findings provide a basis for the in vitro propagation of the specific genotype represented by the single donor tree used in this study. The selected growth regulator concentrations should therefore be regarded as suitable for this genotype under the tested conditions, and their applicability across *C. officinarum* requires validation using additional donor trees and genotypes.

## Figures and Tables

**Table 1:** Effects of culture conditions on the shoot proliferation coefficient and plantlet height of *Camphora officinarum*.

Experiment	Treatment	Proliferation Coefficient	Mean Plantlet Height (cm)
Basal medium screening	MS	4.57 ± 0.50 a	1.79 ± 0.23 a
	Modified MS	1.73 ± 0.23 b	1.24 ± 0.01 b
	WPM	2.03 ± 0.15 b	1.18 ± 0.01 b
Growth regulator combination screening	Z1	2.45 ± 0.23 c	1.56 ± 0.13 bc
	Z2	3.97 ± 0.49 b	1.67 ± 0.07 b
	Z3	6.37 ± 0.66 a	2.01 ± 0.08 a
	Z4	2.63 ± 0.52 c	1.20 ± 0.05 bc
	Z5	2.35 ± 0.61 c	1.38 ± 0.11 cd
Growth regulator concentration optimization	J1	8.16 ± 0.51 a	2.73 ± 0.24 a
	J2	1.53 ± 0.44 d	1.96 ± 0.12 cd
	J3	5.18 ± 1.03 b	2.66 ± 0.10 ab
	J4	1.42 ± 0.07 d	1.76 ± 0.08 d
	J5	4.89 ± 0.15 b	2.33 ± 0.19 b
	J6	4.28 ± 0.30 bc	1.84 ± 0.09 cd
	J7	4.13 ± 1.17 bc	2.02 ± 0.28 bcd
	J8	3.38 ± 1.05 c	2.14 ± 0.12 bc
	J9	1.92 ± 0.52 d	2.09 ± 0.27 bcd

Note: Data are presented as the mean ± standard deviation (SD) of three replicates. Within each experiment, values in the same column that do not share a lowercase letter differ significantly (*p* < 0.05). No significance comparisons were made between experiments. Conditions for basal medium screening and formulations Z1–Z5 are described in Section 4.3; formulations J1–J9 are listed in Table 3.

**Table 2:** Effects of basal media on rooting in *Camphora officinarum*.

Basal Medium	Rooting Rate (%)	Mean Root Number	Mean Root Length (mm)
CK1	51.11 ± 6.94 c	0.83 ± 0.15 c	7.33 ± 2.37 c
WPM	82.22 ± 6.94 ab	2.98 ± 0.74 b	82.62 ± 5.18 a
MS	75.56 ± 13.88 b	1.74 ± 0.55 c	47.32 ± 11.51 b
1/2MS	92.96 ± 1.70 a	4.37 ± 0.41 a	93.09 ± 16.07 a

Note: CK1 contained no basal nutrient formulation. All treatments were supplemented with 0.50 mg/L IBA, 30 g/L sucrose, and 6 g/L agar. Data are presented as the mean ± standard deviation. Values within the same column that do not share a lowercase letter differ significantly (*p* < 0.05).

## Data Availability

The data presented in this study are available within the article and Supplementary Materials.

## Supplementary Materials

The following supporting information can be downloaded at https://www.mdpi.com/article/10.3390/plants15213497/s1: Table S1: Effects of different disinfection treatments on explant culture of *Camphora officinarum*; Table S2: Effects of different plant growth regulator concentrations on axillary bud initiation in *Camphora officinarum*; Table S3: Range analysis and analysis of variance for the orthogonal experiment on shoot proliferation; Table S4: Effects of different transplanting substrates on the survival of *Camphora officinarum* plantlets.
