# Supplementary material for: Physiological and Endogenous Hormonal Responses During In Vitro Culture of Citral-Rich Camphora officinarum
Source: Plants (Basel). 2026 Nov 9;15(21):3497. doi: 10.3390/plants15213497 (PMC13765542; doi:10.3390/plants15213497)
Supplement: Supplementary file 1 [file plants-15-03497-s001.zip › plants-4643528-supplementary.pdf]

# Supplementary Materials

## Physiological and Endogenous Hormonal Responses during In Vitro Culture of Citral-Rich *Camphora officinarum*

**Table S1. Effects of different disinfection treatments on explant culture of *Camphora officinarum*.**

| Experiment | Treatment | Survival rate (%) | Contamination rate (%) | Browning rate (%) | Bud break rate (%) |
|---|---|---|---|---|---|
| Disinfectant combination | 75% ethanol for 30 s + carbendazim (1:1000) for 30 min | 12.22±5.09 b | 83.33±3.34 a | 4.44±7.70 a | 2.22±1.92 b |
| Disinfectant combination | 75% ethanol for 30 s + 20% NaClO for 10 min | 26.67±12.02 b | 64.44±18.36 a | 8.89±8.39 a | 4.44±5.09 b |
| Disinfectant combination | 75% ethanol for 30 s + 0.1% $HgCl_2$ for 7 min | 83.33±3.34 a | 12.22±3.85 b | 4.44±1.93 a | 24.44±5.09 a |
| $HgCl_2$ exposure time | 3 min | 25.56±8.39 b | 74.44±8.39 a | / | 2.22±1.92 b |
| $HgCl_2$ exposure time | 5 min | 43.33±6.67 b | 54.44±5.09 b | 2.22±1.92 c | 5.56±3.85 b |
| $HgCl_2$ exposure time | 7 min | 78.89±5.09 a | 8.89±1.92 d | 12.22±6.94 bc | 43.33±6.67 a |
| $HgCl_2$ exposure time | 10 min | 26.67±3.33 b | 22.22±7.70 c | 51.11±8.39 a | 5.56±1.92 b |

Note: Data are presented as the mean ± standard deviation. Within each experiment, values in the same column that do not share a lowercase letter differ significantly ($p < 0.05$). In the $HgCl_2$ exposure-time experiment, all explants were treated with 75% ethanol for 30 s, followed by 0.1% $HgCl_2$ for the indicated duration. The symbol "/" is retained from the original records and indicates an unreported value, not zero.

**Table S2. Effects of different plant growth regulator concentrations on axillary bud initiation in *Camphora officinarum*.**

| Plant growth regulator | Concentration (mg/L) | Survival rate (%) | Bud break rate (%) | Browning rate (%) |
|---|---|---|---|---|
| 6-BA | 0.50 | 44.44±5.09 bc | 21.11±8.39 cd | 3.33±3.33 c |
| 6-BA | 1.00 | 51.11±5.09 b | 32.22±6.94 b | 6.67±3.33 bc |
| 6-BA | 2.00 | 63.33±3.33 a | 60.00±3.33 a | 8.89±5.09 bc |
| 6-BA | 3.00 | 42.22±5.09 c | 25.56±3.85 bc | 12.22±3.85 b |
| 6-BA | 4.00 | 45.56±3.85 bc | 14.44±5.09 d | 21.11±5.09 a |
| IBA | 0.05 | 61.11±7.64 a | 55.56±10.00 a | 13.33±10.41 a |
| IBA | 0.10 | 45.00±5.00 ab | 15.00±5.00 b | 13.33±5.77 a |
| IBA | 0.50 | 28.33±17.56 b | 10.00±5.00 b | 11.67±7.64 a |
| IBA | 1.00 | 45.00±5.00 ab | 13.33±7.64 b | 20.00±13.23 a |

Note: IBA was maintained at 0.50 mg/L during 6-BA screening, and 6-BA was maintained at 2.00 mg/L during IBA screening. Data are presented as the mean ± standard deviation. Within each screening experiment, values in the same column that do not share a lowercase letter differ significantly ($p < 0.05$).

**Table S3. Range analysis and analysis of variance for the orthogonal experiment on shoot proliferation.**

**A. Range analysis**

| Statistic | Level | 6-BA | NAA | KT |
|---|---|---|---|---|
| K | 1 | 4.87 | 10.48 | 14.21 |
| K | 2 | 12.4 | 9.94 | 9.08 |
| K | 3 | 17.62 | 14.47 | 11.6 |
| R | R | 4.24 | 1.51 | 1.71 |

Note: K is the sum of proliferation coefficients at the corresponding factor level; R is the range of the level means (maximum minus minimum). Levels 1–3 represent increasing concentrations; the concentrations are specified in Section 2.3 of the main text.

**B. Analysis of variance**

| Factor | Sum of squares | df | Mean square | F | p |
|---|---|---|---|---|---|
| 6-BA | 81.990 | 2 | 40.995 | 68.854 | <0.001 |
| NAA | 12.203 | 2 | 6.101 | 10.248 | .001 |
| KT | 13.177 | 2 | 6.589 | 11.066 | .001 |
| Error | 11.908 | 20 | 0.595 | | |

**Table S4. Effects of different transplanting substrates on the survival of *Camphora officinarum* plantlets.**

| Treatment | Substrate composition | Survival rate (%) |
|---|---|---|
| Y1 | Yellow soil | 8.33±7.64 c |
| Y2 | Peat:yellow soil (1:1) | 11.67±7.64 c |
| Y3 | Nutrient soil:yellow soil (1:1) | 30.00±8.66 b |
| Y4 | Peat:perlite (1:1) | 67.78±7.64 a |
| Y5 | Nutrient soil:perlite (1:1) | 35.00±10.00 b |
| Y6 | Vermiculite:perlite:river sand (2:2:3) | 30.00±5.00 b |

Note: Substrate ratios are those used in the experiment. Survival was assessed 30 d after transplanting. Data are presented as the mean ± standard deviation. Values in the same column that do not share a lowercase letter differ significantly ($p < 0.05$).
